# Rambutan seed waste-derived nitrogen-doped carbon dots with l-aspartic acid for the sensing of Congo red dye

**DOI:** 10.1039/d2ra07620a

**Published:** 2023-02-22

**Authors:** Muhammad Zulfajri, Sri Sudewi, Rizki Damayanti, Genin Gary Huang

**Affiliations:** a Department of Chemistry Education, Universitas Serambi Mekkah Banda Aceh Aceh 23245 Indonesia Muhammad.zulfajri@serambimekkah.ac.id; b Department of Pharmacy, Universitas Sam Ratulangi Manado 95115 Indonesia; c Department of Medicinal and Applied Chemistry, Kaohsiung Medical University Kaohsiung 80708 Taiwan

## Abstract

In this study, new nitrogen-doped carbon dots (N-CDs) were prepared by utilizing rambutan seed waste and l-aspartic acid as dual precursors (carbon and nitrogen sources) through a hydrothermal treatment method. The N-CDs showed blue emission in solution under UV light irradiation. Their optical and physicochemical properties were examined *via* UV-vis, TEM, FTIR spectroscopy, SEM, DSC, DTA, TGA, XRD, XPS, Raman spectroscopy, and zeta potential analyses. They showed a strong emission peak at 435 nm and excitation-dependent emission behavior with strong electronic transitions of C=C/C=O bonds. The N-CDs exhibited high water dispersibility and great optical properties in response to some environmental conditions such as heating temperature, light irradiation, ionic strength, and storage time. They have an average size of 3.07 nm and good thermal stability. Owing to their great properties, they have been used as a fluorescent sensor for Congo red dye. The N-CDs selectively and sensitively detected Congo red dye with a detection limit of 0.035 μM. Moreover, the N-CDs were utilized to detect Congo red in tap and lake water samples. Thus, rambutan seed waste was successfully converted into N-CDs and these functional nanomaterials are promising for use in important applications.

## Abstract 2

In this study, new nitrogen-doped carbon dots (N-CDs) were prepared by utilizing rambutan seed waste and l-aspartic acid as dual precursors (carbon and nitrogen sources) through a hydrothermal treatment method and applied as a fluorescent sensor toward Congo red.

## Graphical abstract

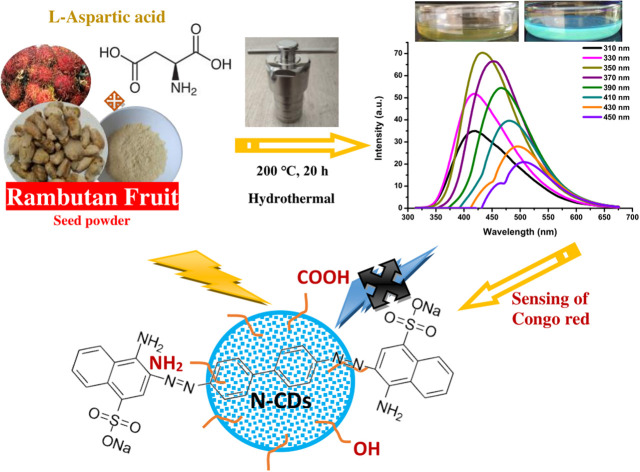

## Introduction

Carbon dots (CDs) are fluorescent carbon-based nanomaterials that were discovered serendipitously during the purification of SWCNTs in 2004.^1^ They are among the amazing materials in the nanocarbon family and are generally discrete and quasi-spherical with a size below 10 nm. CDs commonly produce typical excitation-dependent emission wavelengths and overcome the limitations of toxic heavy metal elements to form nanomaterials.^2^ The CDs are excellent candidates and demonstrate encouraging performances in optoelectronics, sensors, fluorescent inks, nano-catalysts, light-emitting diodes, medical diagnosis devices, drug delivery, and bioimaging due to their unique properties including strong chemical inertness, high anti-photobleaching, great optical characteristics, great chemical stability, excellent photostability, easy functionality, high solubility, low toxicity, high biocompatibility, strong fluorescence properties, and eco-friendliness.^2–5^

Although CDs have many advantages compared to noble metal nanoclusters, organic molecules, and semiconductor quantum dots, their chemical and optical characteristics still require enhancement by tuning the surface and core of their chemical structures.^6^ For fluorescence sensor applications, serious challenges remain for CDs, particularly regarding their lack of selectivity and sensitivity and their low fluorescence quantum yields (QY). Doping heteroatoms such as nitrogen (N), sulfur (S), boron (B), and phosphorous (P), into carbon cores is an effectual means of modulating the intrinsic characteristics of CDs. This can adjust and control the electronic structure, optical, and chemical properties as well as surface chemical groups, thus developing their applications.^7^ Heteroatomic doping is the general method for increasing the fluorescence characteristics of CDs, resulting in a high fluorescent QY of the CDs.^8,9^ Among the various heteroatoms, the N dopant is ideal owing to its proportional atomic size to that of C and it has five valence electrons to interact with C atoms simply.^10^ The N atoms in CDs can supply many electrons and coat the CD surface, thus altering their electronic and optical properties.^11^ The nitrogen atoms can extend the conjugated π-electrons in the carbon skeleton, improving the CDs' QY.^12^

CDs without N-doping have a large HOMO–LUMO gap. N-Doping in CDs reduces the HOMO–LUMO gap so less energy is needed for excitation and the sensing ability is improved.^13^ The few valence electrons of the N dopant encourage chelation with –COOH and –NH_2_ groups present on the surface of CDs, which immediately interact with the functional ligands of the analyte *via* the typical interactions, and lower the HOMO–LUMO gap to improve the sensing performance of CDs.^14^ N-Doped CDs (N-CDs) provide a simple and effective sensor for sensing specific molecules due to the NH_2_ groups that can simplify and increase the interaction with the selective analyte.^2^ Therefore, suitable control of the functional groups, structures, and compositions of CDs is required for developing their sensing applications.^15^

Recently, the preparation of CDs *via* a sustainable chemistry approach has received considerable attention because it decreases both the time and cost, simplifies labor, and produces high yields and eco-friendly materials.^16^ Starting materials for the production of CDs are available, particularly from natural resources. The CDs derived from various starting materials exhibit distinct structures with certain applications.^17,18^ The CDs have different functional groups on the surfaces with great surface area-to-volume ratios, which offer valuable benefits for doping or functionalizing with other small molecules.^19^ The CDs can be synthesized using various methods. Hydrothermal treatment is a facile, low-cost, and green method that produces CDs with a narrow size distribution. To date, a facile synthetic method and a green source for obtaining highly fluorescent CDs are still highly desirable and there is still space to explore cheap and natural starting materials that act as carbon precursors. Green sources work as great candidates for producing fluorescent CDs. Unlike semiconductor quantum dots or metal nanoparticles, CDs can easily be synthesized using a single-step reaction.^20,21^ Natural resources are abundant in carbon, nitrogen, and oxygen in the form of carbohydrates and proteins.^5^ The use of simply obtainable and highly abundant natural sources as starting materials is preferential for the preparation of CDs not only for practical massive production but also for sustainable chemistry purposes.

Several specific advantages of using synthetic dyes over natural dyes include their excellent stability in oxygen, light, and pH, which clarifies the existing trend for the use of synthetic dyes instead of natural dyes.^22^ However, their excessive utilization in foods and textiles may cause harmful health effects on several organs and health systems, such as allergic reactions, migraines, eczema, anxiety, oxidative stress, DNA damage, liver problems, hepatotoxicity, kidney function disease, abnormal lipid profiles, and brain tissue injury.^23^ A very small amount of dye in water is highly visible, and discharging even a small amount of dye into the water can cause serious harmful effects on aquatic organisms and food webs due to the carcinogenic and mutagenic effects of synthetic dyes.^24^ Congo red is the first synthetic dye produced that is capable of dying cotton directly and belongs to the class of protein-binding dyes.^25^ Congo red is an anionic diazo dye prepared by coupling tetrazotised benzidine and two molecules of naphthionic acid. Congo red causes an allergic reaction and is metabolized to benzidine, a human carcinogen.^26^ It is unsafe, toxic, and can cause anaphylactic shock and cancer. Congo red is often used in textile, printing and dyeing, paper, leather, food, cosmetics, rubber, and plastics industries.^27^ The Congo red staining process requires a lot of water and so water pollution occurs.^28^ Due to its complex aromatic structure with high stability, it is resistant to biodegradation and photodegradation.^29^ The environment and human health are thus subjected to various hazards and major risks and, therefore, the levels of color added to products must be accurately quantified. As such, it is urgent and essential to develop and establish a simple, cost-effective, rapid, efficient, and sensitive analytical method to determine Congo red to protect human and environmental health.^30^

**Fig. 1:**
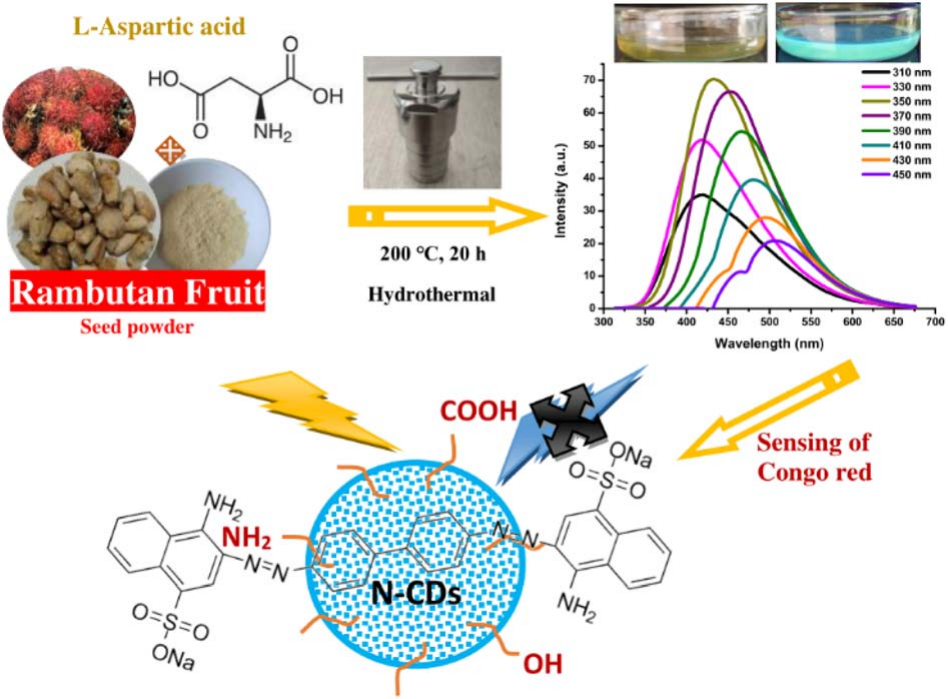
The synthesis, optical properties, and applications of N-CDs.

Recently, numerous efforts have been devoted to developing green synthetic strategies for the production of CDs by exploring various natural resources as starting materials such as milk,^31^ potato starch,^32^ cabbage,^33^ soybeans,^34^ orange juice,^35^ banana juice,^36^ and watermelon peel.^37^ The present work demonstrates, for the first time, the low-cost, facile, and eco-friendly preparation of N-CDs from rambutan seed waste as the carbon source and l-aspartic acid (Asp) as the nitrogen source through the hydrothermal method. The rambutan seeds were collected after being discarded by people after eating the fruit flesh. This rambutan seed powder contains many ingredients that can be converted to CDs, especially carbohydrate content. Amino acids are verified as a good nitrogen source for the synthesis of highly fluorescent N-CDs.^38^ Asp is one of the non-essential amino acids that can improve the fluorescence properties of N-CDs. The synthesized N-CDs through the optimization of the synthesis conditions showed strong fluorescence properties with a high QY. The stability, optical, and physicochemical properties of the N-CDs were explored in detail. The N-CDs could be utilized as a fluorescent sensor for Congo red. The schematic representation of the synthesis, optical properties, and applications of N-CDs is illustrated in Fig. 1.

## Experimental

### Materials and chemicals

Rambutan fruit was purchased from a traditional fruit market at Simpang Barabong, Aceh Besar district, Aceh province, Indonesia. H_2_SO_4_ and HCl were obtained from Honeywell Research Chemicals (Offenbach, Germany) and Avantor-J.T. Baker (Center Valley, PA, USA). Several heavy metal chloride substances including AlCl_3_, BaCl_2_, CaCl_2_·2H_2_O, CdCl_2_, CoCl_2_·6H_2_O, CrCl_2_·6H_2_O, CuCl_2_·2H_2_O, FeCl_2_·4H_2_O, HgCl_2_, KCl, MgCl_2_·6H_2_O, NaCl, NiCl_2_, PbCl_2_, SnCl_2_, SrCl_2_·6H_2_O, and ZnCl_2_ were obtained from Pudak Scientific and Merck. Some synthetic dyes including Brilliant blue (BB), crystal violet (CV), eosin Y (EY), erythrosine (ET), indigosol grey (IG), carmoisine (CM), Congo red (CR), methyl red (MR), methylene blue (MB), Ponceau 4R (P4R), purpurin (PP), Remazol red (RR), rhodamine 6G (R6G), rhodamine B (RhB), rose bengal (RB), Allura red (AR), and eosin B (EB) were purchased from Indocol, Deco-Tatoo, and Merck. NaOH and l-aspartic acid were obtained from Merck. Quinine sulfate was received from Alfa Aesar (Ward Hill, MA, USA). All the chemicals and reagents were of analytical grade and were used as received without further purification. Cellulose ester dialysis membrane with 1000 Da MWCO was obtained from Shanghai Yuanye Bio-Technology Co. Ltd. The pure water (18.2 MΩ cm) was produced by a Millipore Simplicity water purification system (Merck KGaA, Darmstadt, Germany) and used throughout the preparation of solutions in this work and the whole experiments.

### Synthesis of N-CDs

A hydrothermal treatment was used to synthesize N-CDs. In brief, 1.0 g of rambutan seed powder and 0.5 M Asp (10.53% N) dispersed in 30 mL pure water were placed in a Teflon-autoclave and hydrothermally reacted for 20 h at 200 °C. After the reaction, the solution was allowed to cool at room temperature to reduce the temperature of the solution. The successful conversion of seed powder and Asp to N-CDs was clarified by changing their colors to dark brown. To filter the solution and remove the large particles, Whatman filter paper No. 42 was used and centrifugation at 9000 rpm for 30 min was performed, followed by filtration using a 0.22 μm Nylon syringe filter membrane. Subsequently, the N-CDs solution was dialyzed overnight *via* a dialysis bag (1000 MWCO) against pure water and the water was changed every 6 h to eliminate the small molecules. The N-CDs were kept in a dark bottle at 4 °C for this study.

### Calculation of the fluorescence QY

The fluorescence QY of the N-CDs was calculated according to a common formula.^39^ Typically, quinine sulfate was diluted in 0.1 M sulfuric acid as a standard with a QY of 54% and a refractive index (*η*) of 1.33. N-CDs were diluted in pure water (*η* = 1.33). The absorbance value (*A*) was kept at 0.05 at 350 nm and the fluorescence spectrum was obtained on excitation at 350 nm. The integrated fluorescence intensity (*I*) and *A* value of N-CDs were compared to quinine sulfate solution, and the QY was calculated by the formula QY_c_ = QY_q_ × (*A*_q_/*A*_c_) × (*I*_c_/*I*_q_) × (*η*_c_^2^/*η*_q_^2^), where c = CDs, and q = quinine sulfate.

### Characterization methods of N-CDs

Highly fluorescent N-CDs were identified as the best candidates for the sensor. The optical characteristics of the N-CDs were measured and analyzed. The UV-vis spectra were recorded using an Agilent 8453 UV-vis spectrometer and a quartz cuvette of 10 mm path length and 3 mL volume. The fluorescence spectra were obtained using an Agilent Cary Eclipse Fluorescence Spectrophotometer. Various excitation wavelengths were used to determine the highest emission intensity of N-CDs. The N-CDs with a high fluorescence emission intensity were applied as a sensor. All measurements were repeated three times. The TEM images were taken from a Hitachi HT-7700 microscope at an acceleration voltage of 200 kV for observing the particle size distribution and morphology of N-CDs. Raman spectra of N-CDs were obtained using a MicroRaman Spectrometer (ProTrusTech Co. Ltd, Taiwan) at 532 nm laser excitation. The surface functional groups of the N-CDs were examined by an ALPHA FTIR spectrophotometer from Bruker. Zeta potential values were obtained using an ELSZ-2000 Zeta Potential & Particle Size Analyzer. The emission color was observed under UV-light at 365/400 nm. Other instruments were used for material characterization including SEM, DSC, TGA, DTA, and XRD.

### Fluorescence stability of N-CDs

The ionic strength effect was evaluated by mixing several NaCl concentrations (0–1000 mM) into the N-CDs solution. The effects of light radiation with increasing radiation times from 0 to 60 min, storage times from 1 to 30 days, and heating temperatures from 25 to 100 °C on the N-CDs solution were also evaluated. Finally, the impact of pH values from pH 2 to pH 10 on the fluorescence intensity of N-CDs was examined and 0.1 mM HCl and NaOH solutions were used to adjust the pH values. For all experiments, the fluorescence spectra of N-CDs were measured at 350 nm excitation wavelength for comparison.

### Fluorescence sensing of Congo red dye

The interaction between N-CDs and Congo red was investigated at room temperature. For the selective study, N-CDs solution (50-fold, 500 μL) and each dye (500 μL) with a final concentration of 25 μM were mixed in an Eppendorf tube with 3 min of interaction time before fluorescence measurements. Besides, some heavy metal ions at 500 μL were added to the N-CDs solution to observe their effects on the fluorescence intensity of N-CDs. For the sensitive study, the final concentration of the 500 μL Congo red solution of 1–100 μM was added to the N-CDs solution (50-fold, 500 μL). The fluorescence spectra of the N-DS-CDs/Congo red system were obtained on excitation at 350 nm.

### Fluorescence sensing of Congo red in real water samples

The application of N-CDs as a fluorescent sensor toward Congo red in real water samples was also examined. The tap and lake water samples were employed without and with filtration/purification, respectively. The samples were added with the standard Congo red solution to the N-CDs solution to final concentrations of 1, 5, and 10 μM. The fluorescence spectra of the systems were recorded and the formula below was used to obtain the recovery percentages:*R* = [(*C*_c_ − *C*_b_)/*C*_a_] × 100%,“*R*” is the % recovery, “*C*_a_” is the added Congo red level, “*C*_b_” is the real Congo red level without the addition of the standard Congo red, and “*C*_c_” is the found Congo red level after the addition of standard Congo red.^40^

## Results and discussion

### Optical properties of N-CDs

**Fig. 2:**
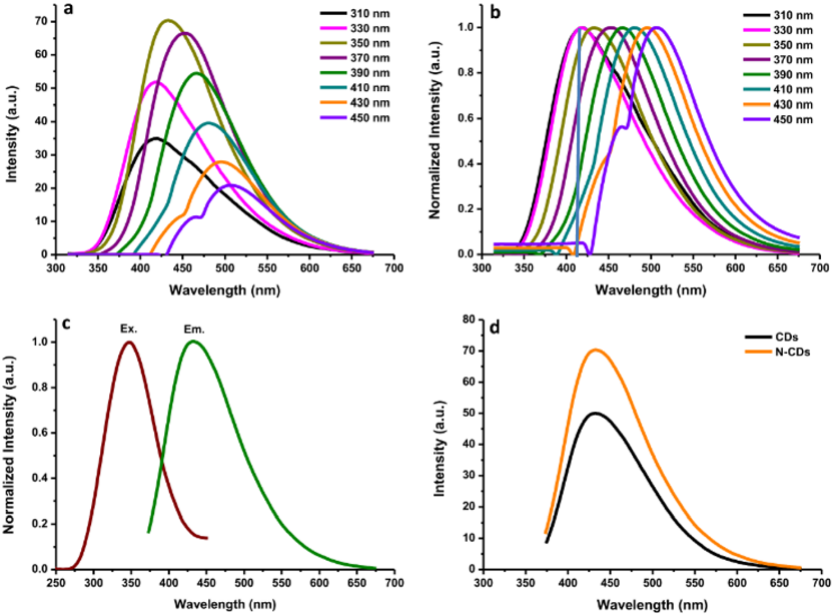
(a) The fluorescence spectra of N-CDs excited with various wavelengths; (b) normalized fluorescence spectra of N-CDs; (c) the optimum excitation and emission spectra of N-CDs, and (d) the comparison of fluorescence spectra of N-CDs and their bare CDs.

The fluorescence and UV-vis spectra of the synthesized CDs were recorded to observe their optical characteristics. The fluorescence spectra of N-CDs excited with various wavelengths are depicted in Fig. 2a, exhibiting unique excitation-based emission behavior. The fluorescence emission peak centers were red-shifted on increasing the excitation wavelength from 300 to 450 nm. As shown in Fig. 2a, the fluorescence emission intensity of N-CDs was gradually enhanced by changing the excitation wavelength from 310 to 350 nm, and subsequently, it gradually decreased with increasing the excitation wavelength to 450 nm. These findings indicate that the fluorescence emission peak centers were red-shifted by altering the excitation wavelength, except for N-CDs with low excitation wavelength (310–330 nm: no red-shift was observed) (Fig. 2b). The optimum excitation and emission spectra of N-CDs are shown in Fig. 2c. The maximum fluorescence intensity of N-CDs was centered at 435 nm (green line) excited at 350 nm (red line). The comparison of the fluorescence spectra of N-CDs and their bare CDs showed an enhancement in the fluorescence emission intensity (Fig. 2d) due to the successful doping of Asp as a nitrogen source for the bare CDs. The fluorescence emission intensity is dependent on the character of the surfaces and most particles of CDs are excited at a specific excitation wavelength.

**Fig. 3:**
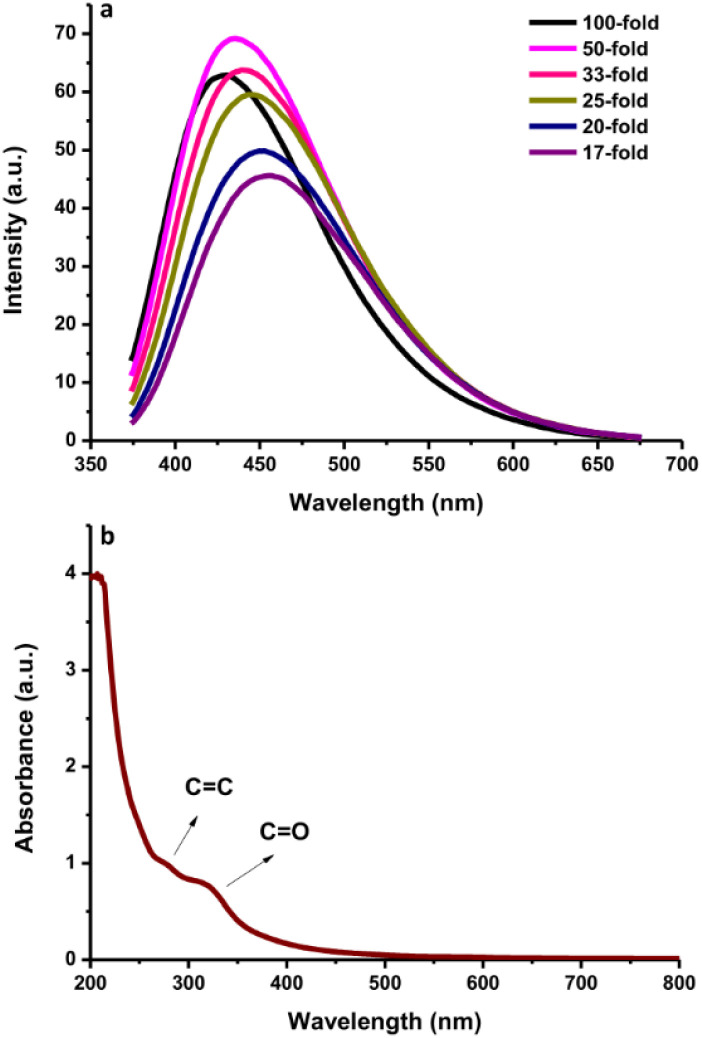
(a) The fluorescence spectra of N-CDs with various dilution levels. (b) The UV-vis absorption spectra of N-CDs.

The fluorescence intensities of N-CDs with various dilution factors are shown in Fig. 3a. The fluorescence emission center was slightly red-shifted by increasing the dilution factor of the N-CDs solution from 17-fold to 100-fold. The highly concentrated N-CDs (17-fold) were separated into monodispersed N-CDs by increasing the amount of pure water to obtain a 50-fold diluted N-CDs solution. The highest fluorescence intensity was observed for the N-CDs with 50-fold dilution when excited at 350 nm. The variation in the emission was attributed to the combination of van der Waals forces to create nanoclusters at a high concentration of N-CDs, leading to increased polarity on the surface of highly concentrated N-CDs.^41^ A high polarity nanocluster might cause various emission behaviors.^42^ After adding more water to increase the dilution beyond the 50-fold N-CDs solution, the emission intensity was reduced due to the excess amount of water in the N-CDs solution. Therefore, the control of the N-CDs levels/dilution factor in solution is valuable for adjusting the emission peak with the highest intensity.

The UV-vis spectra of the N-CDs showed a wide absorption band with a shoulder from 200 to 500 nm (Fig. 3b) and an intense absorption peak at ∼280 nm due to the conjugated C=C bonds with the π–π* energy transition. A shoulder between 300 nm and 500 nm was also observed. This shoulder peak comes from C=O bonds with n–π* energy transition.^16^ Additionally, the fluorescence QY of the N-CDs was 16.87%. The FL QY of N-CDs increased from 10.24% without N-doping. These results indicate that the synthesized N-CDs had typical optical characteristics.

### Physicochemical properties of N-CDs

**Fig. 4:**
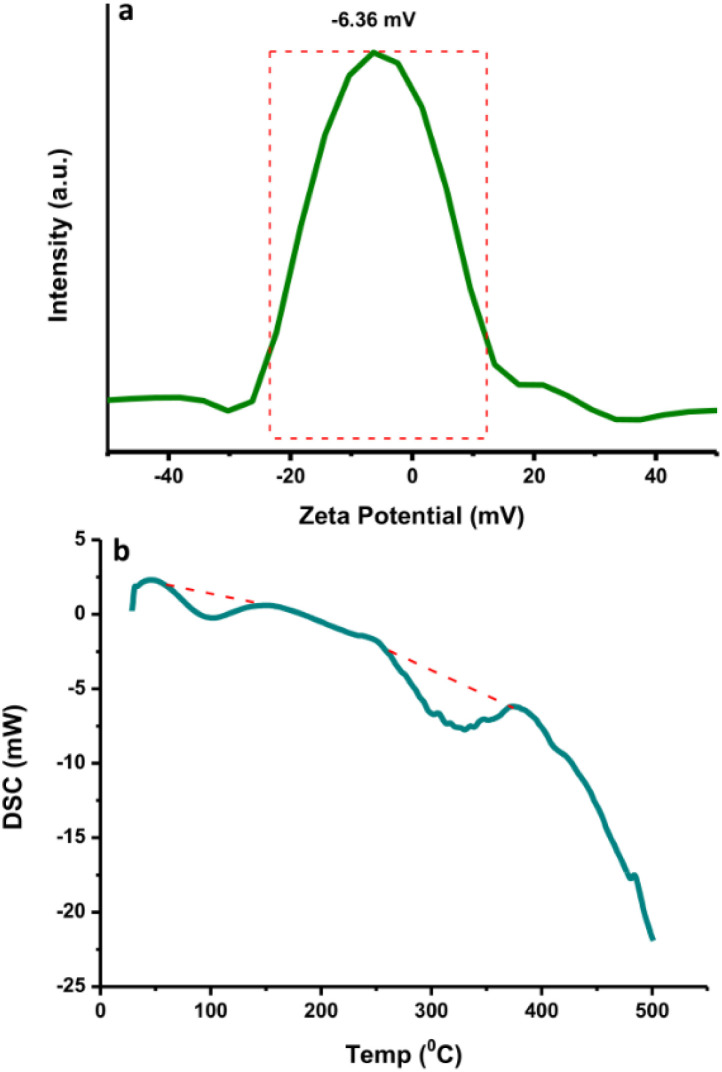
(a) The zeta potential and (b) DSC curve of N-CDs.

The N-CDs formed stable systems in pure water due to their highly hydrophilic nature. To determine their surface charge, the zeta potential was measured and found to be −6.36 mV (Fig. 4a). This value was the center of the broad peak ranging from −20 mV to +10 mV, which was stable. The nanoparticles with zeta potential values from −10 to +10 mV tend to be neutral. The zeta potential value is highly sensitive to the alterations that occur upon dilution, including various pH values and ionic strength levels.^43^ At a pH above 7 (basic condition), the zeta potential is usually very negative, representing stable anions. When the pH is below 7 (acidic condition), the zeta potential value is usually less negative until zero;^43^ afterwards, the zeta potential value becomes more positive in strongly acidic conditions, which represents the positively charged surroundings of the N-CDs. The surface charge is responsible for the simple dispersion and great stability of the CDs system. The CDs system was easily dispersed in pure water and remained stable without precipitation for as long as 30 days at room temperature. DSC analysis was performed to determine whether energy is released or absorbed by N-CDs when treated at high temperatures where the endothermic (heat absorption) and exothermic (heat evolution) processes coupled with the thermal degradation of the obtained N-CDs can occur. DSC was recorded at room temperature up to 500 °C using simultaneous thermal analysis. The DSC curve of anhydrous N-CDs in Fig. 4b shows two endothermic peaks at 100 °C and 300 °C and the complete thermal degradation of the sample occurred simultaneously. The anhydrous N-CDs were monitored to determine the heat absorption phenomenon during the thermal degradation process.

**Fig. 5:**
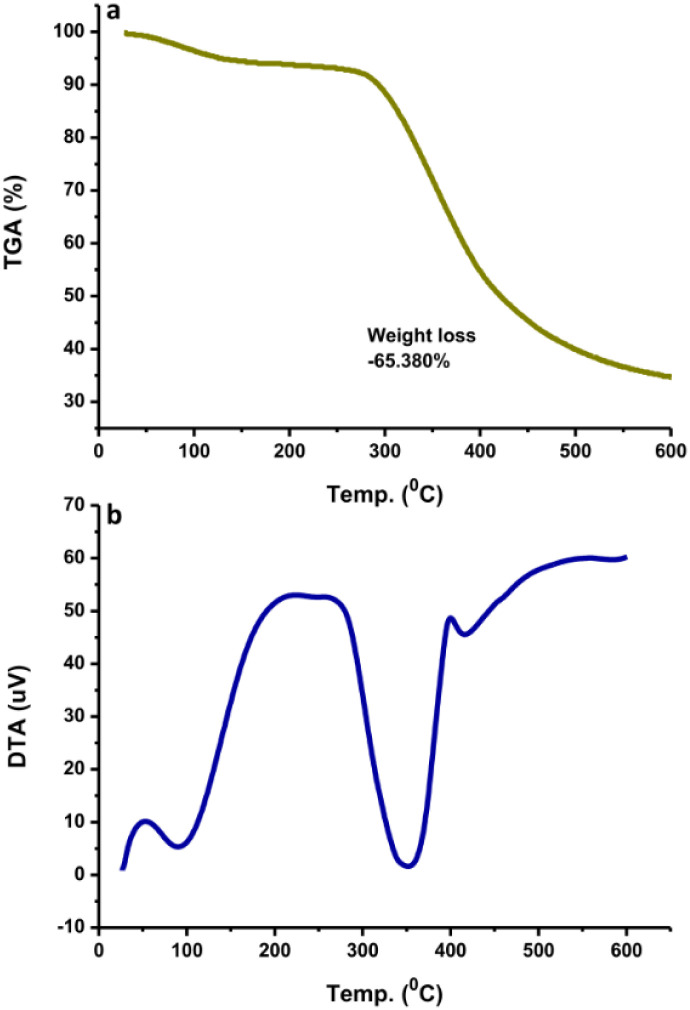
(a) TGA and (b) DTA curves of N-CDs.

TGA measures the amount and rate of change of the material weight (weight loss) with increasing temperature under controlled conditions. The weight loss at different temperatures determines the composition as well as the thermal stability of the sample. TGA analysis showed that the N-CDs slightly decreased in stability until 300 °C and lost weight at a later temperature (Fig. 5a). This may be due to the degradation of the chemical components found on the N-CDs' surface. At 600 °C, N-CDs lost ∼65.38% in weight. The DTA thermogram showed the temperature differences in the sample, where the DTA signal is defined as a function of temperature. The exothermic peak of N-CDs has maximum temperatures of 100 °C and 350 °C (Fig. 5b). Another exothermic process was observed at 425 °C after the temperature increased to 600 °C where the thermogram increased. The thermogram did not increase after raising the temperature to 600 °C.

**Fig. 6:**
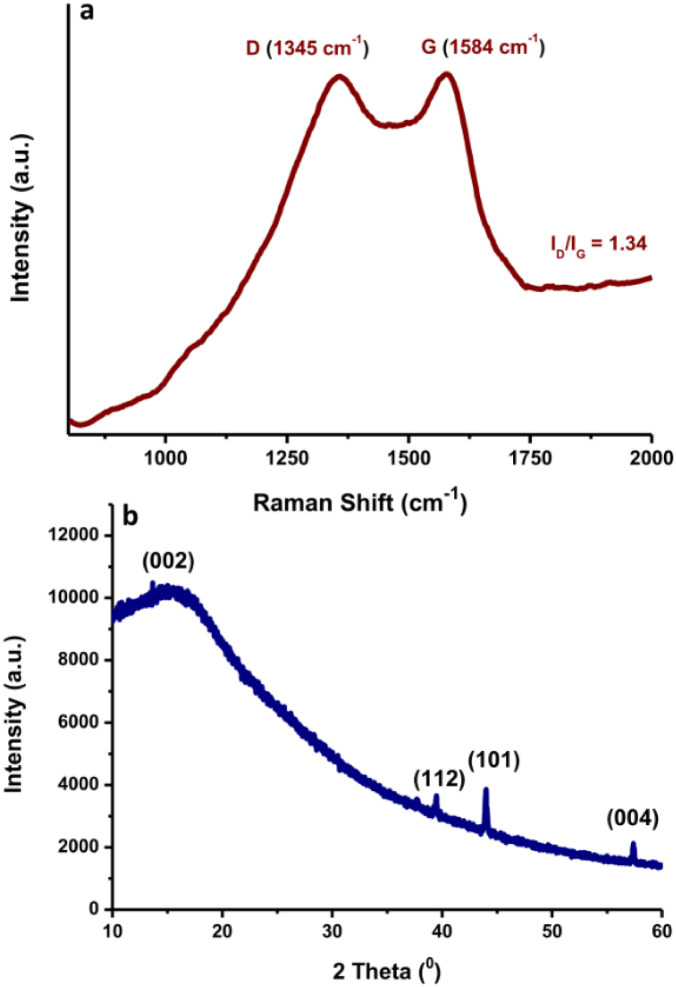
(a) Raman spectrum and (b) XRD pattern of N-CDs.

Raman spectroscopy with laser excitation at 532 nm was also utilized to characterize the intrinsic structure of the N-CDs. The Raman spectrum of N-CDs in the region of 800–2500 cm^−1^ is included in Fig. 6a. The Raman spectra were tilted up because of the intense background signal induced by the strong fluorescence properties of N-CDs. The peaks positioned at *ca.* 1345 cm^−1^ and 1584 cm^−1^ are associated with the typical D and G bands of carbon nanomaterials, respectively. The D-band reflects the disorder or defects of sp^3^ carbon atoms, while the G band indicates sp^2^ carbon networks with in-plane vibrations on the surface structure of N-CDs.^44^ It was observed that N-CDs have an *I*_D_/*I*_G_ ratio of *ca.* 1.34. The N-CDs showed a wider D band, indicating that the intercalation of N atoms into the conjugated carbon backbone caused disordered structures.^45^ These findings suggest that the graphitic structure of N-CDs was destroyed during the doping of nitrogen elements in the presence of a larger fraction of defects. The typical XRD pattern of the prepared N-CDs is shown in Fig. 6b. A broad peak from 10° to 20° centered at 2*θ* = 17° was observed. The presence of the broad peak followed by a weak peak indicated the amorphous form of the N-CDs. The diffraction peak centered at 2*θ* = 44° was assigned to the diffraction pattern 101 of graphite carbon. It is worth mentioning that the shape attests to the existence of a micrographic structure in the N-CDs.^46^

**Fig. 7:**
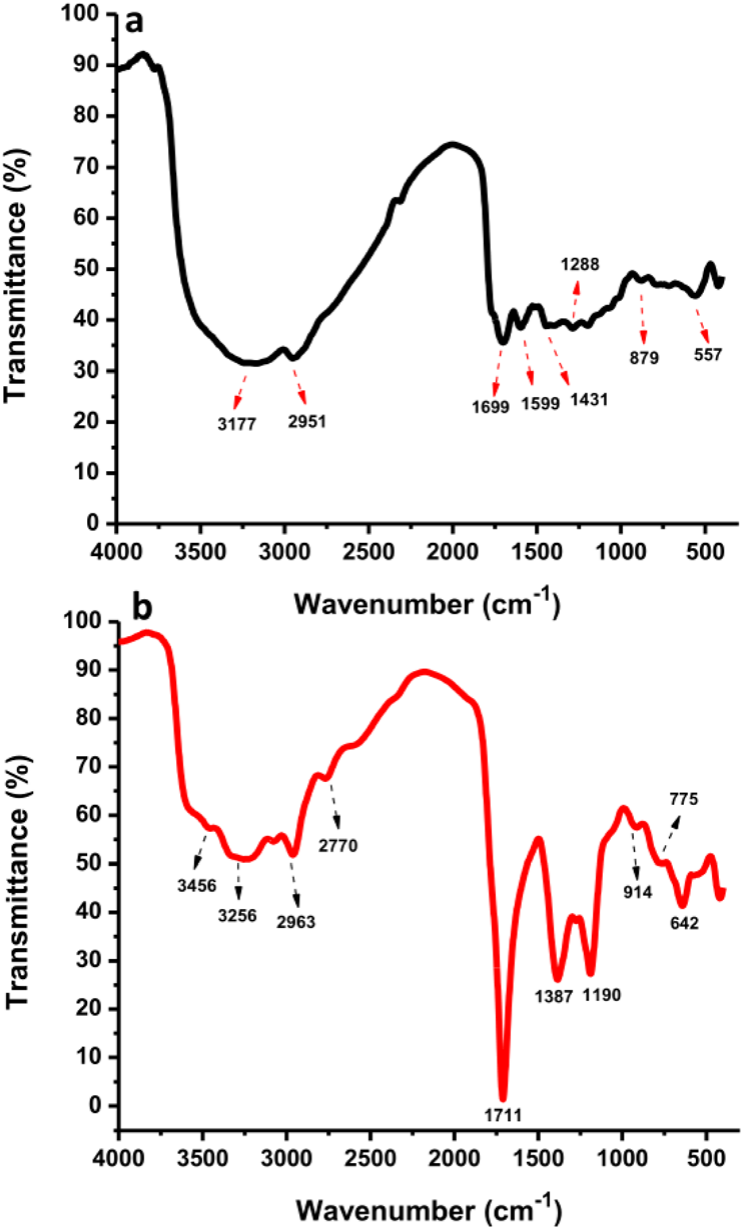
The FTIR spectra of (a) CDs and (b) N-CDs.

The surface functional groups of CDs and N-CDs were investigated by FTIR spectroscopy (Fig. 7a and b). The FTIR spectrum of the CDs showed the presence of O–H and C–H at 3177 cm^−1^ and 2951 cm^−1^ while the N-CDs' spectrum showed peaks at 3256 cm^−1^ and 2963 cm^−1^, respectively.^47,48^ From Fig. 7b, the band at 2770 cm^−1^ was correlated to C–H stretching vibrations.^49^ The stretching vibrations of N–H at 3456 cm^−1^ were monitored in the high regions from N-CDs. The bands at 1699 cm^−1^ and 1599 cm^−1^ were associated with the C=O and C=C stretching of CDs while bands at 1711 cm^−1^ were associated with the C=O/C=C stretching of N-CDs.^50^ The –COOH groups were observed at 1431 and 1387 cm^−1^ for CDs and N-CDs, respectively.^51^ The peak at 1387 cm^−1^ also corresponds to C–N bending vibrations. The peak at 1288 cm^−1^ corresponds to O–H groups from CDs. The peak at 1190 cm^−1^ corresponds to C–O–C/C–N stretching vibrations.^52^ Other peaks at 642 cm^−1^ and 914/775 cm^−1^ were identified as C–H stretching vibrations and O–H stretching/NH_2_ wagging vibrations from N-CDs. The bands at 879 cm^−1^ and 557 cm^−1^ were associated with O–H stretching and C–H bending vibrations from CDs. These findings indicate that N-CDs have amino, hydroxyl, carbonyl, and carboxylic groups originating from organic moieties in rambutan seed powder and Asp after hydrothermal treatment. These functional groups endowed the N-CDs with good water solubility, without aggregation or loss of fluorescence properties after one month.

**Fig. 8:**
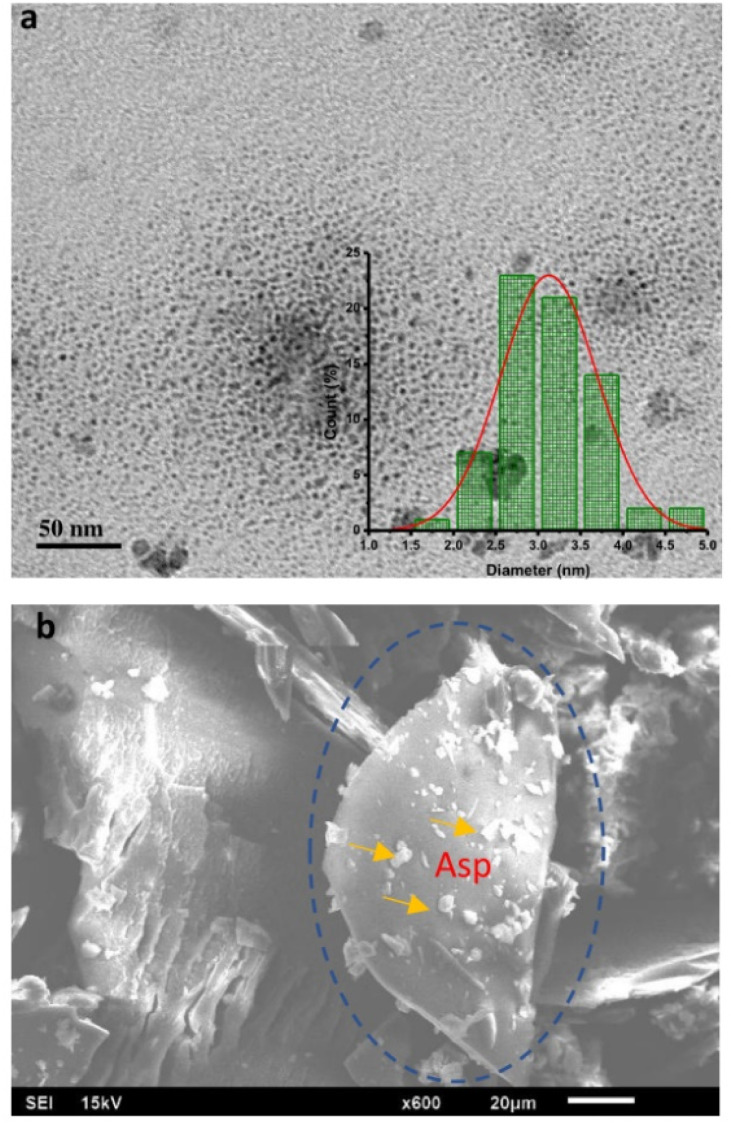
(a) the TEM and (b) SEM images of N-CDs.

The morphological evaluation of the N-CDs was accomplished by TEM and SEM. The images of N-CDs were used to characterize their structure, size, and shape. The TEM image with scale bars of 50 nm exhibited that N-CDs were monodisperse and spherical over all with uniform distribution in the range of 1.52–4.61 nm as represented in the histogram in the inset in Fig. 8a. The average size of N-CDs was calculated to be 3.07 nm. Fig. 8b shows the SEM morphology of the N-CDs. The surface of N-CDs was successfully doped by nitrogen element in Asp. The N-CDs showed many nitrogen dopants on their surface.

### Fluorescence stability of N-CDs

**Fig. 9:**
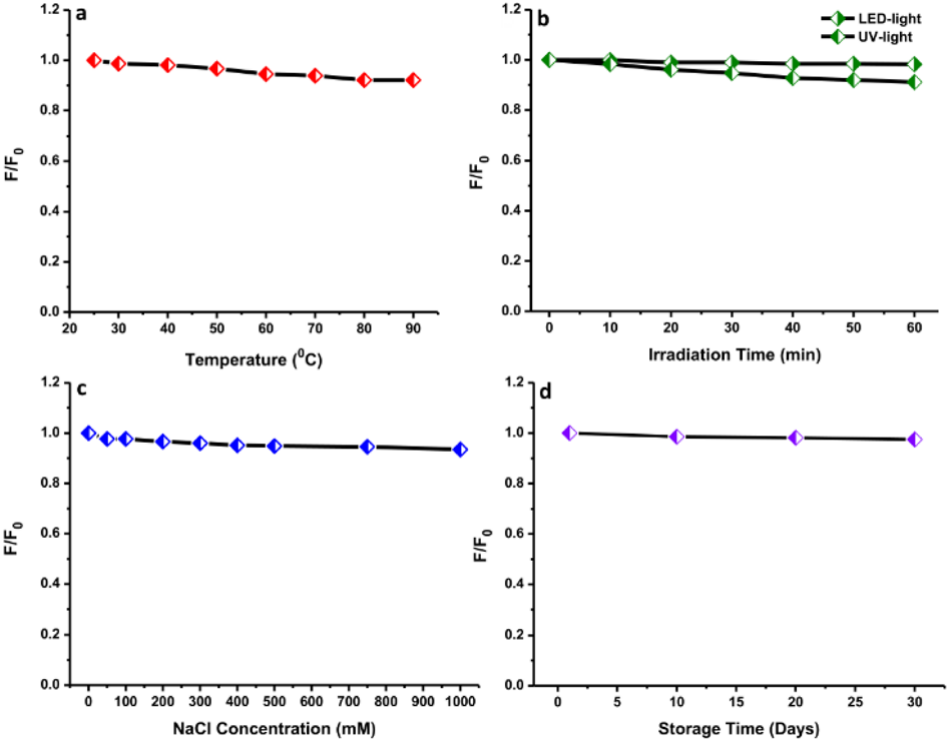
The effects of different (a) heating temperatures, (b) light radiation times, (c) ionic strengths, and (d) storage times on the fluorescence spectra of N-CDs.

The evaluation of the stability of the N-CDs solution based on their fluorescence characteristics under numerous environmental conditions was carried out since the environmental states can affect the performance of N-CDs. The thermal stability of N-CDs was evaluated by heating them at 25–90 °C. The temperature range from room temperature to near the boiling point of water was selected, where much water can be removed from N-CDs solution between 90 °C and 100 °C. Therefore, 90 °C was selected as a high temperature. There was a small effect on the fluorescence emission intensity after heating at a high temperature (90 °C) (Fig. 9a). After heating, the fluorescence emission intensity was slightly reduced, indicating that the heating did not permanently destroy the structure or surface functional groups of N-CDs. The functional groups containing oxygen or nitrogen atoms could avoid particle aggregation at an elevated temperature, preserving the stability of N-CDs.^53^ Furthermore, the fluorescence intensity of the N-CDs solution was monitored after irradiating with LED light and UV light (Fig. 9b). The fluorescence emission intensity of N-CDs after irradiating under LED light remained constant without perturbation even after irradiating for 1 h. Meanwhile, the fluorescence emission intensity of N-CDs was reduced by about 7% after being irradiated under UV light for 1 h. Hence, to maintain the fluorescence properties of N-CDs, the N-CDs solution must be stored in dark bottles and exposure to UV-light must be avoided.

The influence of the ionic concentration from salt on the fluorescence characteristics of N-CDs was evaluated by mixing with different concentrations of NaCl solution to ensure their solution stability (Fig. 9c). The fluorescence emission intensity exhibited a neglectable reduction with the addition of a salt solution containing ions at 1000 mM. This event indicated that the N-CDs were still stable even at an elevated ionic level. The stability of N-CDs at a high ionic level led to the assumption that no ionization of the surface functional groups occurred.^54^ The electrostatic interaction showed a low disturbance of the fluorescence properties of N-CDs. All results indicated that the N-CDs have overall high stability of their fluorescence properties. The shelf-life of N-CDs was also investigated by keeping the N-CDs solution at room temperature. There were no significant perturbations observed in the emission intensity after storing for 30 days, indicating their longer shelf-life and great water stability (Fig. 9d). The N-CDs solution exhibited a homogeneous state over a long time without precipitation.

**Fig. 10:**
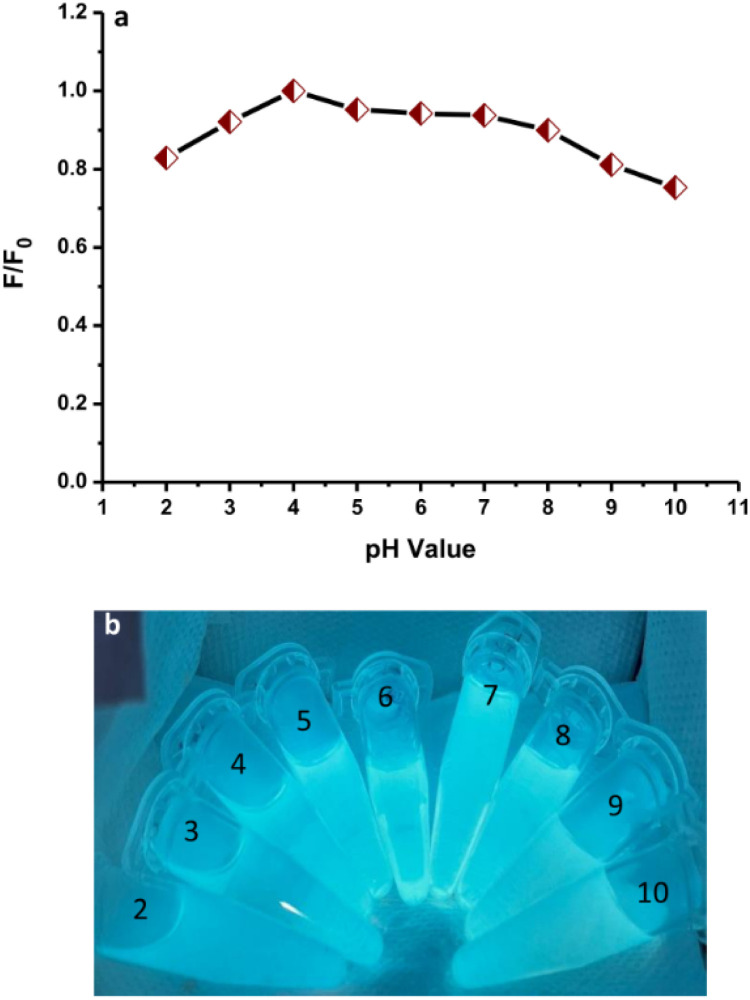
(a) The effect of pH on the fluorescence spectra of N-CDs. (b) The N-CD solutions with different pH values under UV-light irradiation.

The fluorescence emission intensities of N-CDs solutions with varied pH values from 2 to 10 were recorded to examine the effects (Fig. 10a). The fluorescence emission intensity of N-CDs increased on adjusting the pH from 2 to 4. Subsequently, the fluorescence emission intensity was slightly reduced but insignificant from pH 5 to 7, indicating that the N-CDs solution was more stable in weakly acidic and neutral conditions. The fluorescence emission intensity of N-CDs was reduced from a pH of 8 to 10. The maximum fluorescence emission intensity of the N-CDs solution was observed at pH 4. The perturbation in the fluorescence emission intensity can be correlated to the protonation or deprotonation activities associated with different functional groups on the N-CDs surface.^55^ The bright blue color of the solutions under UV-light irradiation was observed for all samples at different pH values (Fig. 10b). This result was correlated with the maintained fluorescence properties of the N-CDs solution at these pH values. The pH values influenced the fluorescence properties but without effects that were too significant.

### Fluorescence sensing of Congo red

**Fig. 11:**
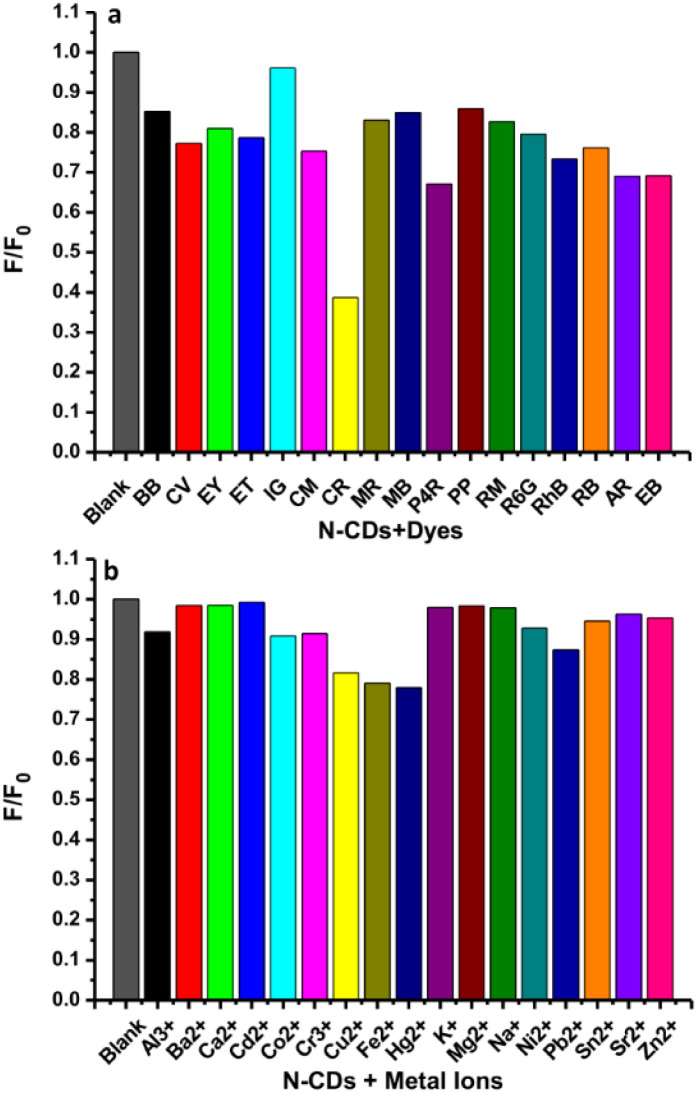
The relative fluorescence intensities (*F*/*F*_0_) of N-CDs solutions without and with various (a) dyes (25 μM) and (b) metal ions (500 μM).

To study the selectivity of this method, the variations in the fluorescence intensity in the presence of different dyes at 25 μM were observed under the same conditions as used for the Congo red. The histogram shows the plot of *F*/*F*_0_*versus* the identical amounts of dyes (Fig. 11a). *F* and *F*_0_ are the fluorescence intensities of N-CDs with and without the analytes. The fluorescence of N-CDs was not significantly reduced by the dyes, except for Congo red. Hence, the N-CDs are forthcoming materials for sensing Congo red as compared to other synthetic dyes. Furthermore, to study the selectivity of this sensor, it is important to examine the fluorescence perturbation of N-CDs by different metal ions. The sensing potential was assessed by observing the initial fluorescence emission intensity of N-CDs for notable variations while adding different representative metal ions. The fluorescence emission spectra of N-CDs solutions mixed with various metal ions (500 μM) were recorded and the alterations in the fluorescence emission intensity at 435 nm were monitored. These metal ions induced various effects on the fluorescence spectra of N-CDs. Fig. 11b shows the selectivity plot of N-CDs with metal ions. The plot of the histogram exhibited *F*/*F*_0_*versus* the identical levels of metal ions. Several heavy metal ions (Cu^2+^, Fe^2+^, and Hg^2+^) can reduce the fluorescence intensity of N-CDs, but not so significantly. Therefore, the N-CDs were not suitable for the selective detection of the representative metal ions.

**Fig. 12:**
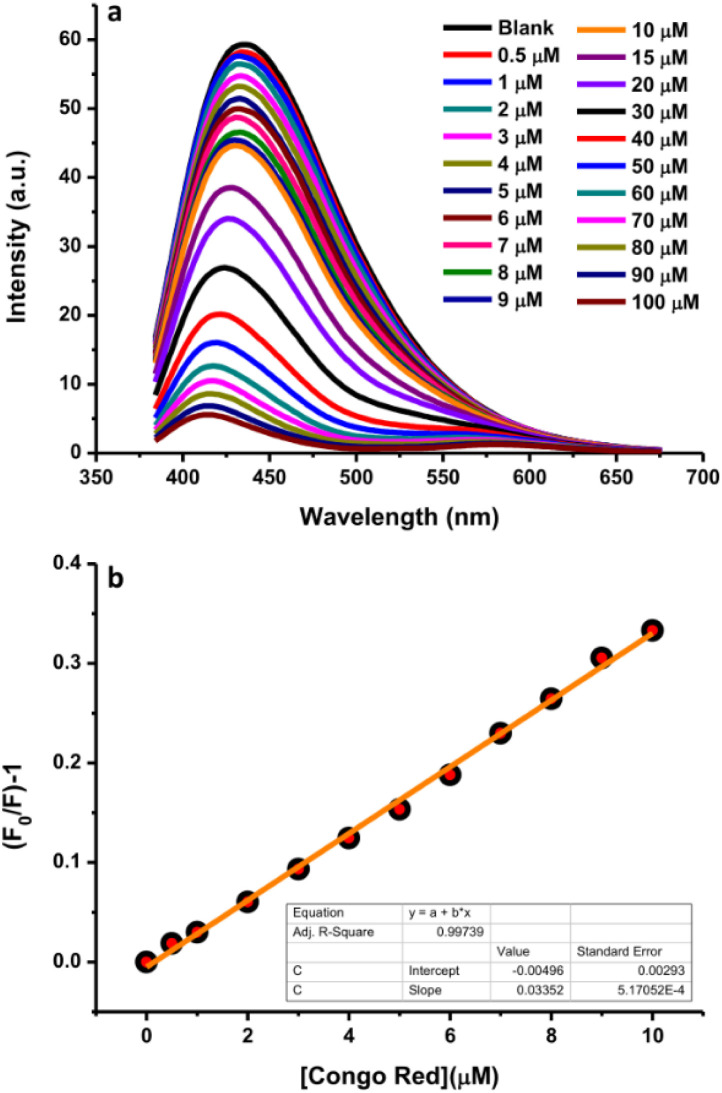
(a) The fluorescence emission intensity quenching of N-CDs in the Congo red concentration range of 0–100 μM. (b) The linear relationship between the fluorescence quenching of N-CDs and Congo red concentration (0–10 μM).

Congo red is one of the most highly utilized dyes for textile colorants. Its excessive content in textile products may result in health problems in the human body due to its carcinogenic effects because of the presence of aromatic amine groups; therefore, its detection is important. For this purpose, a highly sensitive and simple analytical method based on the fluorescence quenching efficiency using N-CDs as the sensing material was adopted. Various concentrations of dye were mixed into the system to monitor the changes in fluorescence intensity. The fluorescence quenching efficiency of N-CDs in the presence of different concentrations of dye (0–100 μM) was investigated and the fluorescence spectra are presented in Fig. 12a. The fluorescence intensities of N-CDs decreased with the increasing dye concentration. A linear relationship was observed for the Congo red concentrations in the range of 0.5–10 μM in the standard curve of the plot of the relational change in the fluorescence intensity (*F*/*F*_0_) and Congo red concentrations (Fig. 12b). The plot exhibited a good linear relationship with a correlation coefficient (*R*^2^) of 0.99739 and the LOD was calculated to be 0.035 μM. Table 1 presents a comparison of the experimental results of several methods with this sensor for the detection of Congo red. Until now, very few studies have focused on the detection of the Congo red dye. This sensor can serve as an alternative method for the sensing of the Congo red dye since the N-CDs can provide an economical, simple, rapid, and sensitive method as compared to other methods.

**Table 1:** Comparison of several methods for the sensing of Congo red

No.	Methods	LOD (μM)	Linear range (μM)	Ref.
1	GO–GCE	0.24	0.01–0.2	56
2	AgNPs	0.86	1.15–345	57
3	Fly Ash (FA)	7.18	7.2–43.1	58
4	N-CDs	0.035	0–10	This study

### Sensing of Congo red in real water samples

To explore the feasibility and significance of applicability in real water samples, this fluorescence sensor was employed to detect Congo red in lake and tap water samples. No Congo red was observed in the water samples on monitoring the fluorescence emission spectra. Therefore, the standard addition technique was adopted. Various amounts of Congo red were spiked into the lake and tap water samples and added to the N-CDs solution, and then the proposed fluorescence responses were recorded. This was performed three times for each sample. The average analysis is shown with the relative standard deviations (RSDs). The fluorescence intensity of the N-CDs was quenched by different concentrations of Congo red standard solution, as shown in Table 2. The recovery percentages ranged between 99.38 and 100.80% for tap water and between 98.40 and 102.15% for lake water samples. The RSDs with low percentages ranged from 0.51 to 1.60% for all water samples, proving that this sensor has good precision and accuracy for sensing Congo red in environmental water samples.

**Table 2:** Sensing results of Congo red in tap and lake water samples (*n* = 3)

Water samples	Added (μM)	Measured (μM)	Recovery (%)	RSD (%)
Tap water	1	0.99 ± 0.01	99.38	0.75
	5	5.02 ± 0.03	100.33	0.51
	10	10.08 ± 0.04	100.80	0.67
Lake water	1	0.98 ± 0.01	98.40	0.92
	5	5.04 ± 0.04	100.86	1.15
	10	10.22 ± 0.13	102.15	1.60

### Possible sensing mechanisms of Congo red

**Fig. 13:**
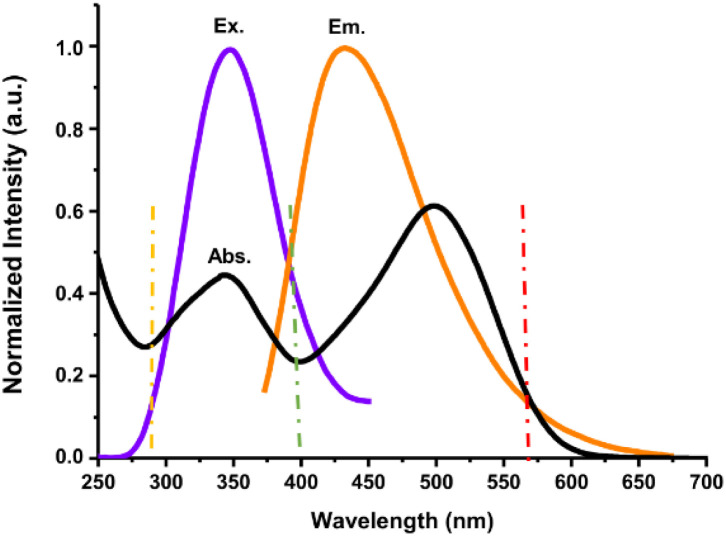
The overlap of the normalized UV-vis spectrum of Congo red (black line) and fluorescence excitation (purple line)/emission (orange line) spectra of N-CDs.

The quenching of the fluorescence emission intensity of N-CDs was evident with the increase in the Congo red concentration. To elucidate the possible sensing mechanisms, the fluorescence spectra of N-CDs and UV-vis absorbance of Congo red were obtained and analyzed. The specific selectivity of N-CDs for Congo red could be clarified by the overlap between the excitation and/or emission spectra of N-CDs (donor) and the absorbance of Congo red (acceptor). The fluorescence emission quenching is probably related to the inner filter effect (IFE). IFE can occur commonly when the absorbance of the quencher overlaps with the excitation and/or emission spectra of the sample. The N-CDs exhibited excitation and emission spectra in the wavelength range of 310–450 nm and 420–507 nm, respectively (Fig. 2a and b). The excitation (purple line) and emission (orange line) wavelengths reached the maximum values of 350 and 435 nm, respectively (Fig. 13). Congo red showed two broad absorption peaks between 275 nm and 600 nm, and the maximum absorption peaks were centered at 345 nm and 500 nm (Fig. 13, black line). The absorption peaks of Congo red almost completely overlapped with the excitation and emission spectra of N-CDs, suggesting that the sensing mechanism is related to the IFE.^59^ Generally, the fluorescence intensities must be corrected if the IFE exists. Thus, the quenching of the emission intensity of N-CDs might be due to the IFE. Meanwhile, the quenching of fluorescence intensity caused by the IFE is temperature independent. As shown in Fig. 9a, there was no temperature effect on the fluorescence emission intensity, denoting that the fluorescence emission quenching is coming from the IFE.^60^

**Fig. 14:**
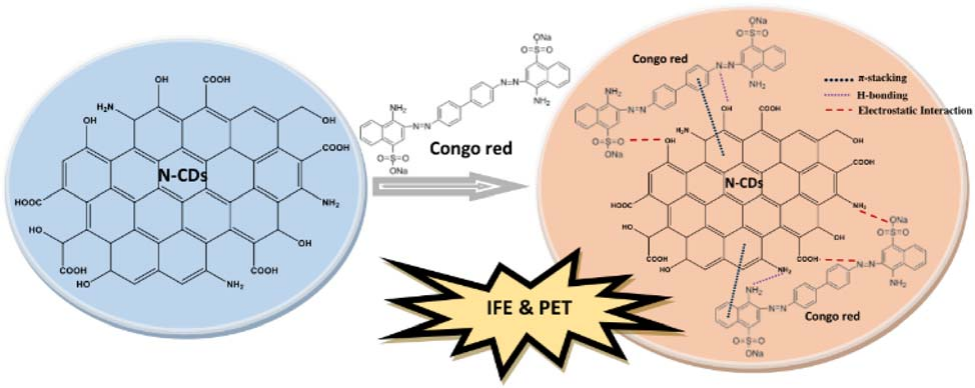
The possible sensing mechanisms of Congo red by N-CDs.

The presence of abundant –OH, –COOH, and –NH_2_ groups on the N-CDs can play a decisive role in the occurrence of intermolecular interactions such as hydrogen bonding and electrostatic interactions with Congo red and consequently result in emission intensity quenching.^61,62^ The electrostatic interactions between functional groups of N-CDs and Congo red allow for hydrogen bonding, which leads to the formation of a non-fluorescence ground state complex.^63^ The hydrogen bonding could occur between –N=N– groups of Congo red and –OH groups of N-CDs, and between NH_2_ groups of both samples. The electron transfer from N-CDs to Congo red led to the fluorescence quenching of the N-CDs. The electron-deficient Congo red caused the fluorescence quenching of N-CDs, indicating that the photoinduced electron transfer (PET) is also the quenching mechanism for the detection.^64^ In addition, the fluorescence quenching may be associated with the effectual interaction of π–π stacking between N-CDs and Congo red. The hydrogen bonding between N-CDs and Congo red can strengthen the π–π stacking between the framework of N-CDs with the benzene ring of Congo red.^65^ π–π stacking interactions occur with the electron transfer from the N-CDs framework to the benzene ring of Congo red. Congo red as the electron acceptor has the propensity to combine with π-donor points on the framework of N-CDs.^66^ The strong π–π stacking interaction is a driving force that brings N-CDs and Congo red close to each other, resulting in easy electron transfer between them.^67^ The stronger the π–π stacking interaction, the more effective the PET process between N-CDs and Congo red. Hence, hydrogen bonding, electrostatic interactions, and π–π stacking interactions easily induced intermolecular electron transfer and resulted in the valuable quenching of the fluorescence emission intensity of N-CDs in the presence of Congo red. The schematic illustration of N-CDs for the sensing of Congo red with their sensing mechanisms can be viewed in Fig. 14. Therefore, N-CDs revealed high sensitivity and selectivity for Congo red, which is associated with the IFE and PET mechanisms.

## Conclusions

The successful conversion of rambutan seed waste into N-CDs *via* carbonization in a simple hydrothermal method has been demonstrated. Rambutan seeds can serve as a carbon source to produce green CDs. Asp was used as a nitrogen dopant to increase the fluorescence properties of CDs. The N-CDs exhibited great optical properties and good stability under various environmental conditions. The N-CDs were quasi-spherical with an average size of 3.07 nm. The fluorescence QY of N-CDs was 16.87%. The fluorescence characteristics of the N-CDs were successfully utilized to sense the Congo red dye *via* the fluorescence quenching mechanism. A fast, selective, and sensitive method for the sensing of Congo red was carried out with an LOD as low as 0.035 μM. Overall, the current study has presented the conversion of a biomass waste resource into a functional nanomaterial and it was successfully utilized for sensing applications.

## Conflicts of interest

There are no conflicts to declare.

## Supplementary Material

## References

[cit1] Xu X., Ray R., Gu Y., Ploehn H. J., Gearheart L., Raker K., Scrivens W. A. (2004). J. Am. Chem. Soc..

[cit2] Zhang X., Chen Y., Ding S. N. (2017). Sci. Bull..

[cit3] Bandi R., Gangapuram B. R., Dadigala R., Eslavath R., Singh S. S., Guttena V. (2016). RSC Adv..

[cit4] Atchudan R., Edison T. N. J. I., Aseer K. R., Perumal S., Karthik N., Lee Y. R. (2018). Biosens. Bioelectron..

[cit5] Vandarkuzhali S. A. A., Jeyalakshmi V., Sivaraman G., Singaravadivel S., Krishnamurthy K. R., Viswanathan B. (2017). Sens. Actuators, B.

[cit6] Park Y., Yoo J., Lim B., Kwon W., Rhee S. W. (2016). J. Mater. Chem. A.

[cit7] Wang Y., Meng Y., Wang S., Li C., Shi W., Chen J., Wang J., Huang R. (2015). Small.

[cit8] Hu Y., Zhang L., Li X., Liu R., Lin L., Zhao S. (2017). ACS Sustainable Chem. Eng..

[cit9] Du Y., Guo S. (2016). Nanoscale.

[cit10] Liu W., Cui Y., Li T., Diao H., Wei S., Li L., Chang H., Zhang B., Wei W. (2018). Chem. Lett..

[cit11] Liu X., Liu J., Zheng B., Yan L., Dai J., Zhuang Z., Du J., Guo Y., Xiao D. (2017). New J. Chem..

[cit12] Liu W., Diao H., Chang H., Wang H., Li T., Wei W. (2017). Sens. Actuators, B.

[cit13] Yi Z., Li X., Zhang H., Ji X., Sun W., Yu Y., Liu Y., Huang J., Sarshar Z., Sain M. (2021). Talanta.

[cit14] Sahu Y., Hashmi A., Patel R., Singh A. K., Susan M. A. B. H., Carabineiro S. A. C. (2022). Nanomaterials.

[cit15] Zhi S., Yang X., Yao C. (2022). Green Anal. Chem..

[cit16] Ansi V. A., Renuka N. K. (2018). Sens. Actuators, B.

[cit17] Li H., Kang Z., Liu Y., Lee S. T. (2012). J. Mater. Chem..

[cit18] Zhu S., Song Y., Zhao X., Shao J., Zhang J., Yang B. (2015). Nano Res..

[cit19] Wang X., Gao T., Yang M., Zhao J., Jiang F. L., Liu Y. (2019). New J. Chem..

[cit20] Ding H., Wei J. S., Xiong H. M. (2014). Nanoscale.

[cit21] Choi C. H., Park S. H., Woo S. I. (2012). ACS Nano.

[cit22] Llamas N. E., Garrido M., Di Nezio M. S., Band B. S. F. (2009). Anal. Chim. Acta.

[cit23] Amin K. A., Abdel Hameid H., Abd Elsttar A. H. (2010). Food Chem. Toxicol..

[cit24] Crini G. (2006). Bioresour. Technol..

[cit25] Purkait M. K., Maiti A., DasGupta S., De S. (2007). J. Hazard. Mater..

[cit26] Chatterjee S., Lee D. S., Lee M. W., Woo S. H. (2009). Bioresour. Technol..

[cit27] Vimonses V., Lei S., Jin B., Chow C. W. K., Saint C. (2009). Chem. Eng. J..

[cit28] Zare K., Sadegh H., Shahryari-Ghoshekandi R., Maazinejad B., Ali V., Tyagi I., Agarwal S., Gupta V. K. (2015). J. Mol. Liq..

[cit29] Han R., Ding D., Xu Y., Zou W., Wang Y., Li Y., Zou L. (2008). Bioresour. Technol..

[cit30] Xu H., Yang X., Li G., Zhao C., Liao X. (2015). J. Agric. Food Chem..

[cit31] Wang D., Zhu L., McCleese C., Burda C., Chen J. F., Dai L. (2016). RSC Adv..

[cit32] Qiang R., Yang S., Hou K., Wang J. (2019). New J. Chem..

[cit33] Alam A. M., Park B. Y., Ghouri Z. K., Park M., Kim H. Y. (2015). Green Chem..

[cit34] Wang S., Sun W., Yang D. S., Yang F. (2020). Beilstein J. Nanotechnol..

[cit35] Sahu S., Behera B., Maiti T. K., Mohapatra S. (2012). Chem. Commun..

[cit36] De B., Karak N. (2013). RSC Adv..

[cit37] Zhou J., Sheng Z., Han H., Zou M., Li C. (2012). Mater. Lett..

[cit38] Xu Y., Wu M., Liu Y., Feng X. Z., Yin X. B., He X. W., Zhang Y. K. (2013). Chem.–Eur. J..

[cit39] Zulfajri M., Gedda G., Chang C., Chang Y., Huang G. G. (2019). ACS Omega.

[cit40] Zulfajri M., Dayalan S., Li W. Y., Chang C. J., Chang Y. P., Huang G. G. (2019). Sensors.

[cit41] Liu H., Zhao X., Wang F., Wang Y., Guo L., Mei J., Tian C., Yang X., Zhao D. (2017). Nanoscale Res. Lett..

[cit42] Issa M. A., Abidin Z. Z., Sobri S., Rashid S., Mahdi M. A., Ibrahim N. A., Pudza M. Y. (2019). Nanomaterials.

[cit43] Pudza M. Y., Abidin Z. Z., Rashid S. A., Yasin F. M., Noor A. S. M., Issa M. A. (2020). Nanomaterials.

[cit44] Song L., Cui Y., Zhang C., Hu Z., Liu X. (2016). RSC Adv..

[cit45] Li Y., Zhao Y., Cheng H., Hu Y., Shi G., Dai L., Qu L. (2012). J. Am. Chem. Soc..

[cit46] Elanthamilan E., Sriram B., Rajkumar S., Dhaneshwaran C., Nagaraj N., Princy Merlin J., Vijayan A., Wang S. F. (2019). Mater. Res. Bull..

[cit47] Li L., Yu B., You T. (2015). Biosens. Bioelectron..

[cit48] Yang X., Zhuo Y., Zhu S., Luo Y., Feng Y., Dou Y. (2014). Biosens. Bioelectron..

[cit49] Oyeleke G. O., Abdulazeez I. A., Adebisi A. A., Oyekanmi K. N., Akinbode S. O. (2021). Org. Polym. Mater. Res..

[cit50] Li L. S., Jiao X. Y., Zhang Y., Cheng C., Huang K., Xu L. (2018). Sens. Actuators, B.

[cit51] Liu W., Diao H., Chang H., Wang H., Li T., Wei W. (2017). Sens. Actuators, B.

[cit52] Bandi R., Dadigala R., Gangapuram B. R., Guttena V. (2018). J. Photochem. Photobiol., B.

[cit53] Khan W. U., Wang D., Wang Y. (2018). Inorg. Chem..

[cit54] Zhang H., Chen Y., Liang M., Xu L., Qi S., Chen H., Chen X. (2014). Anal. Chem..

[cit55] Soni H., Pamidimukkala P. S. (2018). Mater. Res. Bull..

[cit56] Shetti N. P., Malode S. J., Malladi R. S., Nargund S. L., Shukla S. S., Aminabhavi T. M. (2019). Microchem. J..

[cit57] Sahraei R., Farmany A., Mortazavi S. S., Noorizadeh H. (2012). Toxicol. Environ. Chem..

[cit58] Harja M., Buema G., Bucur D. (2022). Sci. Rep..

[cit59] Chen S., Yu Y. L., Wang J. H. (2018). Anal. Chim. Acta.

[cit60] Xiao N., Liu S. G., Mo S., Li N., Ju Y. J., Ling Y., Li N. B., Luo H. Q. (2018). Talanta.

[cit61] Nie Y., Liu Y., Su X., Ma Q. (2019). Microchem. J..

[cit62] Liu S., Yang H., Yang S., Huang Y., Xiang Z., Ouyang G. (2020). Anal. Chim. Acta.

[cit63] Chan K. K., Yap S. H. K., Yong K. T. (2018). Nano-Micro Lett..

[cit64] Zulfajri M., Rasool A., Huang G. G. (2020). New J. Chem..

[cit65] Das A., Biswas S. (2017). Sens. Actuators, B.

[cit66] Chaudhary S., Kumar S., Mehta S. K., Umar A., Ajmal Khan M. (2019). Chem. Phys. Lett..

[cit67] Ansi V. A., Vijisha K. R., Muraleedharan K., Renuka N. K. (2020). Sens. Actuators, A.

